# SNW1 Orchestrates BMP Signaling in Early Embryonic Patterning

**DOI:** 10.1371/journal.pbio.1001018

**Published:** 2011-02-15

**Authors:** Caitlin Sedwick

**Affiliations:** Freelance Science Writer, San Diego, California, United States of America; PLoS, United States of America

**Figure:**
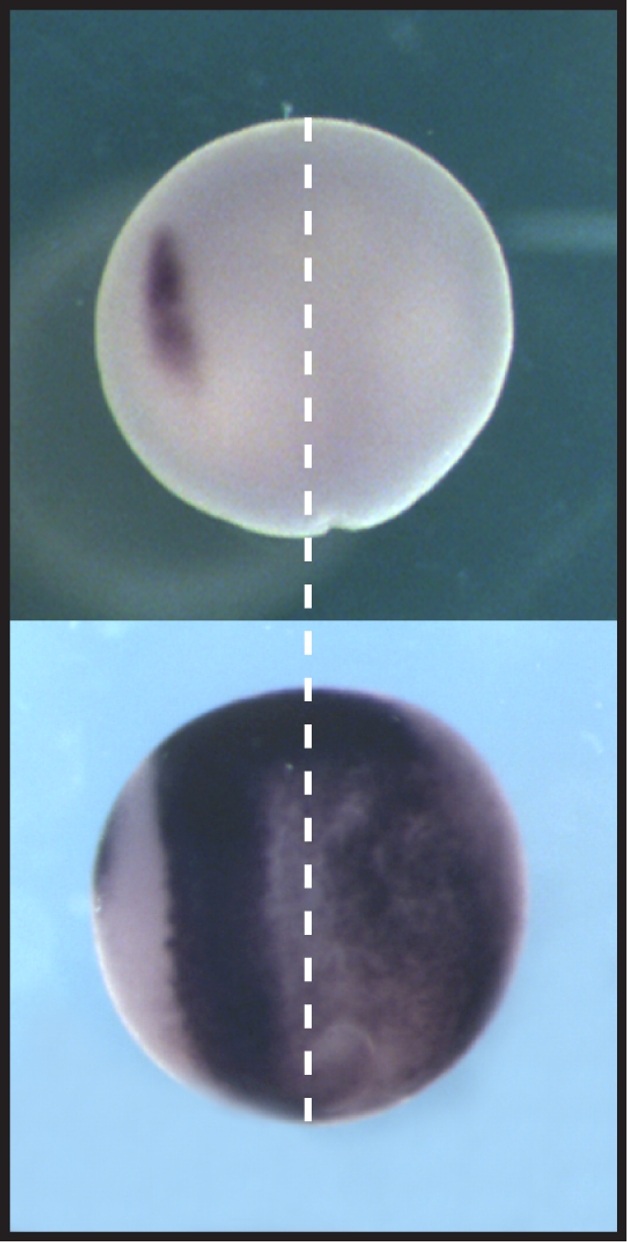
Creating sharp borders: SNW1 orchestrates the signaling required for neural plate border and neural crest formation (left). Loss of SNW1 in frog embryos inhibits both developmental events (right).

To determine their proper developmental path during embryonic development, cells rely on positional cues obtained from interactions with neighboring cells, plus other external signals provided by gradients of secreted proteins known as morphogens. The bone morphogenetic proteins (BMPs) are one family of morphogens that help direct this early embryonic patterning. After the process known as gastrulation, which establishes the embryo's inner, middle, and outer tissue layers, BMP gradients further drive the differentiation of the outermost layer into the neural plate, neural crest, and epidermal tissues. The neural plate later develops into the nervous system, while the neural crest gives rise to the bones and cartilage of the face and to other specialized tissues. How the BMP gradients that drive these events are themselves established, however, has remained mysterious, until now. In this issue of *PLoS Biology*, Mary Wu, Caroline Hill, and colleagues uncover a protein that controls BMP activity in these early tissues.

Through a functional screen in frog embryos (an organism in which early development is easily observed), Wu and colleagues set out to identify factors needed for development of the neural crest. Their screen turned up a protein, SNW1, which, when overexpressed, prevented proper development of the neural crest. For more insights into how SNW1 affects development, the group looked more closely at where and when it is expressed in embryos. They found that, immediately after fertilization, embryos can rely on maternally supplied SNW1 protein and mRNA that was stored in the egg. But, once an embryo has completed gastrulation, its cells start making their own SNW1. SNW1 is expressed throughout the embryo, but it is strongly enriched on the dorsal side of the embryo, in the region of flattened, thickened tissue that makes up the neural plate.

To find out what SNW1 does during embryonic development, the authors injected frog and zebrafish embryos with synthetic antisense oligonucleotides that prevent production of SNW1 protein. Embryos can use maternally supplied SNW1 until gastrulation, so the antisense SNW1 injection had no effect until after that stage. But after gastrulation, the treatment caused a blurring of the normally sharp border that distinguishes the neural plate from surrounding tissues. Also, while in normal embryos the neural crest develops adjacent to the neural plate, the antisense-injected embryos failed to generate neural crest tissues at all.

What accounts for the developmental defects observed in the treated embryos? As BMPs are required for both neural plate border specification and neural crest development, the authors examined whether perturbing the expression of SNW1 affects BMP signaling in embryos. The group showed that BMP signaling activity is depressed in antisense-injected embryos, and elevated in embryos that overexpress SNW1. Either manipulation severely impaired embryonic development, indicating that SNW1-mediated regulation of BMP activity is important for proper embryonic development.

One way that SNW1 might affect BMP activity is by controlling where BMP signaling takes place in embryos. To find out where BMP signaling happens in post-gastrulation embryos, Wu and colleagues examined zebrafish embryos that produce a fluorescent protein wherever BMP signaling is active. They discovered that BMP activity is normally concentrated in a tight horseshoe-shaped band that runs around the anterior edge and sides of the neural plate. Loss of SNW1 expression in the neural plate causes a strong drop in BMP signaling levels within this band of cells, and when BMP is lost here, neural crest tissue does not form.

Collectively, the authors' experiments show that when SNW1 is lost in the neural plate, the border between the neural plate and neighboring tissues is blurred, and simultaneously, the horseshoe-shaped BMP band that abuts it is also lost. Without these two developmental landmarks, the neural crest—which normally arises at the junction between these two regions—fails to develop. SNW1 is therefore an important factor in determining proper embryonic development after gastrulation. And, although the exact mechanism by which SNW1 modulates BMP activity as yet remains unclear, the identification of this connection opens new doors to a better understanding of embryonic development.

**Wu MY, Ramel M-C, Howell M, Hill CS (2011) SNW1 Is a Critical Regulator of Spatial BMP Activity, Neural Plate Border Formation, and Neural Crest Specification in Vertebrate Embryos. doi:10.1371/journal.pbio.1000593**

